# Teen pregnancy in the setting of familial dilated cardiomyopathy: a case report

**DOI:** 10.1186/s12884-022-04427-2

**Published:** 2022-02-01

**Authors:** Joshua S. George, Jeffrey Johnson

**Affiliations:** Department of Obstetrics and Gynecology, Wayne State University, Detroit, USA

**Keywords:** Familial Dilated Cardiomyopathy, Teen Pregnancy, Prenatal Care

## Abstract

**Background:**

Women with pre-existing forms of familial cardiomyopathy are at increased risk for morbidity and mortality due to hemodynamic changes of pregnancy. There is a lack of consensus about the management and care for these patients given the rarity of this condition. This case represents possibly the youngest pregnant familial dilated cardiomyopathy patient to deliver and the youngest patient to be fitted for a wearable cardiac defibrillator in the postpartum period.

**Case Presentation:**

A 14-year-old gravida 1 with familial dilated cardiomyopathy presented late for prenatal care at 38 weeks, which precluded typical care plans including baseline and serial echocardiograms, medication management, and routine prenatal care. An echocardiogram showed severely decreased left ventricular systolic function compared to studies from one year prior. Three days later the patient presented in labor and had a spontaneous vaginal delivery complicated by postpartum hemorrhage. Her postpartum course was notable for persistence of decreased cardiac function testing and placement of a wearable cardiac defibrillator for prevention against life threatening arrhythmias.

**Conclusion:**

This case report adds to the literature on pregnancy complicated by familial dilated cardiomyopathy and describes management best practices and considerations during the antepartum, intrapartum, and postpartum periods.

## Background

Pregnancy and the postpartum period are a time of hemodynamic instability. These changes can be consequential for the morbidity and mortality of women with pre-existing cardiomyopathy (CDM), a subset of which is familial dilated cardiomyopathy (FDC) [1, 2]. Pregnancy has been shown to worsen cardiac function and outcomes in patients with CDM, especially in those with left ventricular ejection fraction (LVEF) < 40% [3, 4]. Pregnancy- induced hypervolemia results in increased end-diastolic dimension and cardiac decompensation. Another contributing factor is the discontinuation of typical management methods for CDM such as aldosterone antagonists and angiotensin converting enzyme inhibitors (ACEI) both of which are recommended to be stopped prior to conception [5]. Other hypothesized etiologies include immune system activation by fetal micro-chimerism and by genetic predispositions that are exacerbated by hemodynamic stress [6]. We present a case of the youngest reported patient with FDC to successfully deliver and describe management considerations based on existing literature.

### Case presentation

A 14-year-old gravida 1 with familial dilated cardiomyopathy initially diagnosed at 15 months presented extremely late to prenatal care at 38 weeks gestation.

### Medical history

The patient was diagnosed at 15 months with dilated cardiomyopathy, small ventricular septal defect (2 mm), and mild mitral regurgitation. Echocardiogram showed mildly increased left ventricular end diastolic diameter at 36 mm and “mildly decreased” ejection fraction (no numerical value was given). The patient was started on enalapril 5 mg twice daily and followed with serial echocardiograms.

The patient had routine clinical evaluation and echocardiograms twice yearly and remained asymptomatic until loss of follow up at age 13. At this time, which was about one year prior to her pregnancy, the patient had exercise stress testing which was normal. Cardiology documentation specified that the patient should not participate in competitive athletics.

### Family history

**Fig. 1:**
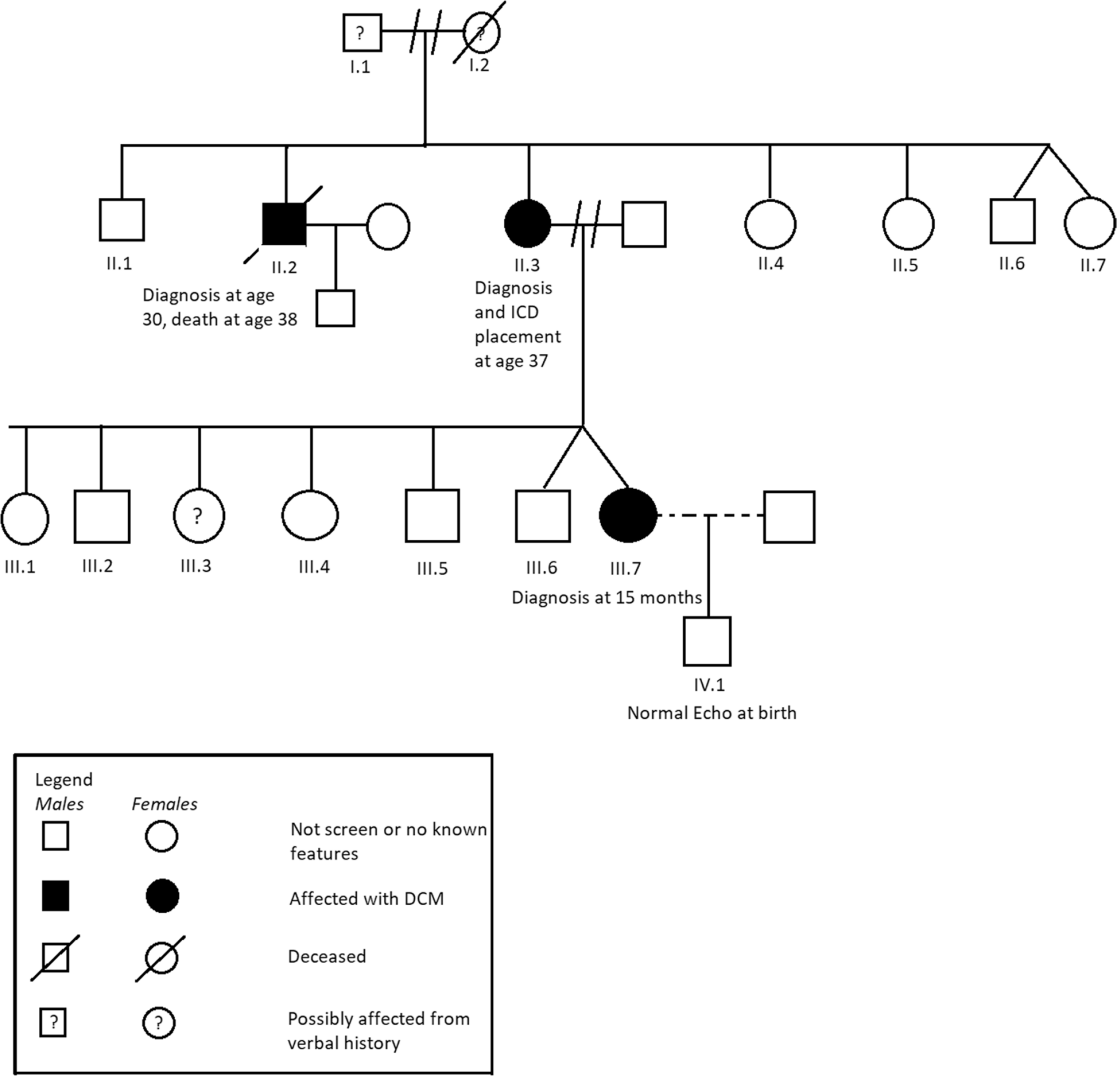
Pedigree of familial dilated cardiomyopathy. III.7 is our patient of interest. (DCM, dilated cardiomyopathy, ICD, intracardiac defibrillator)

The patient’s maternal uncle was diagnosed with CDM at age 30 and died at age 39. The patient’s mother was diagnosed with CDM and atrial fibrillation and underwent ICD placement at age 37. She had declined genetic testing. The patient’s other maternal uncles and aunts have declined symptoms and genetic testing as well. The maternal grandmother developed heart failure and passed in her sixties and the maternal grandfather is still alive and was recently diagnosed with heart failure. The patient’s mother denied any cardiac disease on the patient’s paternal side. The patient’s twin brother was born with cleft lip and palate but the mother reports his cardiac testing was normal. The patient had five older siblings, some of whom have had cardiac symptoms but have declined testing (Fig. 1).

### Initial presentation

The patient was lost to follow up for one year until time of presentation. She was brought in by her mother after she noted increased lower extremity and abdominal swelling and had a positive home pregnancy test. The patient stated that she thought her last period was the previous July and stated that intercourse was consensual. She did not have any cardiac related symptoms upon presentation. Her physical exam was normal with cardiovascular exam reported as normal rate and rhythm without murmur or pulmonary findings. EKG showed normal sinus rhythm. A bedside ultrasound dated the pregnancy at 37 weeks and 6 days. Her prescribed medications were enalapril 5 mg twice daily, furosemide 20 mg daily, aspirin 81 mg daily, spironolactone 25 mg twice daily, metoprolol 25 mg 1/2 pill per day; however, the patient reported poor adherence to the regimen. Rapid follow up was arranged and the patient was instructed to discontinue the enalapril and the spironolactone.

The patient presented to the Maternal Fetal Medicine (MFM) Clinic two days later. A maternal echocardiogram revealed increased LVEDD, ventricular septal defect, and severely decreased LVEF of approximately 20% as compared to 42% from one year prior. (Table 1).

### Labor presentation

Three days later, the patient presented in active labor. Her initial cervical examination was 5 cm dilation/80% effacement/ 0 station and she was contracting every 2–3 min. Fetal heart tracing was Category 1. She reported shortness of breath with contractions only and denied any chest pain or palpitations. Physical exam was normal and EKG showed normal sinus rhythm. All vital signs were within normal limits.

MFM recommendations included close monitoring of fluid status with administration of Lasix if fluid overload was noted, maintenance of normal heart rate to allow for diastolic filling, epidural analgesia administration to alleviate patient pain, and assisted second stage delivery with vacuum or forceps as clinically indicated. They also recommended slow administration of epidural analgesia to minimize the risk of sympathetic blockade and acute drop in systemic venous return.

### Delivery

The patient had a spontaneous vaginal delivery three hours later of a male infant weighing 3275 g, with APGAR scores of 8 and 9 at 5 and 10 min, respectively. The second stage of labor lasted 20 min. Delivery was complicated by postpartum hemorrhage caused by deep second degree perineal laceration and atonic uterus. Laceration repair and uterine massage were conducted. Postpartum oxytocin 10U, misoprostol 1000mcg rectally, carboprost 0.2 mg IV, and tranexamic acid 1 g IV were each administered once following institutional postpartum hemorrhage protocols. Total estimated blood loss was 1474 cc. Thorough exam was conducted and uterus was noted to be firm and laceration hemostatic.

### Postpartum

The postpartum course was complicated by acute blood loss anemia. Starting hemoglobin was 10.4 mg/dL and hemoglobin on postpartum day one was 8.1 mg/dL (Table 2). Pt reported shortness of breath with ambulation so pediatric cardiology recommendations were for transfusion of one unit packed red blood cells along with 10 mg furosemide. The patient’s intake and output were closely monitored to achieve as close to net neutral as possible. Post transfusion hemoglobin was 8.8 mg/dL and the patient reported resolution of symptoms. The patient was counseled on breastfeeding but declined, deciding to exclusively formula feed her infant.

The patient was restarted on her prior medication regimen on postpartum day one as well as prophylactic subcutaneous heparin. On postpartum day two, the patient was fitted for a Zoll LifeVest (LifeVest®, ZOLL, Pittsburgh, PA, USA) wearable cardiac defibrillator (WCD). By postpartum day three, the patient was meeting all milestones and was discharged home. She received Depo-Provera for contraception prior to discharge.

At one month postpartum, the patient had an echocardiogram which showed severely dilated left ventricle with insignificant change from time of delivery and moderately to severely decreased ejection fraction, which was noted to be slightly improved from the time of delivery. The patient still had her WCD on and reported no symptoms and was meeting all postpartum milestones. She received a Paragard intrauterine device (ParaGard; Teva Pharmaceutical Industries Ltd., Sellersville, PA, USA) for long-acting reversible contraception and was dispositioned to further follow up and management with pediatric cardiology.

At four months postpartum, the patient had follow-up with pediatric cardiology and was still asymptomatic and able to walk several blocks without symptoms. She was designated as New York Heart Association Class II Heart Failure. Repeat echocardiogram showed persistently severely dilated left ventricle with no further change from prior study and moderately to severely decreased ejection fraction with no change from one month postpartum. She was recommended to continue with WCD use with consideration for permanent ICD placement based on symptomatology and echocardiographic findings.

### Neonatal care

The patient delivered a male infant with normal genitalia. A postnatal echocardiogram identified normal four chamber intracardiac anatomy with normal atrioventricular and ventriculoarterial relationships.

## Discussion and conclusions

Our patient met criteria for FDC, defined as idiopathic dilated cardiomyopathy in two or more family members [7]. This condition is distinguished from peripartum cardiomyopathy (PPCM) which has various definitions but all of which mention the absence of pre-existing heart disease as a criterion [6, 8]. However, Van Spaendonck et al. raise the possibility that a subset of PPCM may be part of the spectrum of FDC with initial presentation in the postpartum period [9]. They demonstrated cases of PPCM in FDC families as well as previously undiagnosed FDC in families with patients with PPCM that did not recover fully. This theory has been substantiated at the genetic level by Ware et al. who showed that 16% of PPCM patients carry FDC-associated gene variants often in the sarcomere protein titin [10]. For previously asymptomatic FDC patients, the hemodynamic challenges of pregnancy may represent the inciting factor that tips a patient into overt disease [9]. Further family history screening and genetic counseling for those PPCM patients that do not recover fully may prove beneficial in early detection of disease in asymptomatic family members. This approach proved successful in prior case reports in the literature [11, 12].

FDC inheritance typically follows an autosomal dominant pattern and often involves cytoskeleton or sarcomere proteins, although a wide variety of genes and inheritance patterns have been described [13]. The American College of Cardiology recommends clinical screening and cascade genetic testing for asymptomatic relatives of index patients as it may improve outcomes and decrease hospitalization and death due to heart failure [14]. If familial mutations are known, prenatal or preimplantation genetic screening can be considered; however, these are not routinely performed due to variable expressivity and cost barriers, among others [6]. Patient autonomy is also a primary consideration in this discussion. Our patient’s mother indicated that some of her siblings and some of the index’s patient’s siblings have had cardiac symptoms but have declined testing, stating that they do not want the intrusion of a medical diagnosis on their lives. While patient wishes should be respected, asymptomatic family members should be thoroughly counseled to notify health care providers of this strong family history as well as cardiac warning signs and symptoms, especially if considering pregnancy.

Known FDC does allow providers an advantage in that proper care can ideally be delivered from the preconception period onward. This was not applicable in our case due to late onset of prenatal care. Fortunately, despite sub-optimal prenatal care and follow up, our patient successfully delivered a healthy child with minimal intrapartum or postpartum complications. This circumstance provides an opportunity to highlight the importance of contraception counseling upon the onset of menses, more likely to be encountered by pediatric providers for these patients. The American Association of Pediatrics recommends that adolescent contraceptive needs be addressed in the primary care setting [15]. However, the proportion of providers who discuss contraception with patients is varying [16].

For an adolescent to seek out preconception counseling is unlikely as in our case, but if applicable, FDC patients would benefit from this counseling [17]. If pregnancy is found in the first or second trimester, appropriate care would involve a discussion with the patient of the risks and benefits of continuation of the pregnancy based on her clinical status and her management options. Regardless of decision, at initial diagnosis of pregnancy, medication reconciliation is necessary. In our case, the patient was not compliant with her regimen of enalapril, furosemide, aspirin, spironolactone, and metoprolol, a potential explanation for the lack of observed adverse effects. Of these medication classes, ACEI are contraindicated due to risks of teratogenicity, fetal renal failure, and neonatal death and spironolactone is not advised due to anti-androgen activity leading to feminization of males in limited animal studies [6]. Diuretics and selective beta blockers such as metoprolol are noted to be relatively safe and can be considered after weighing maternal and neonatal risks and benefits [18]. There are no specific imaging guidelines but a baseline echocardiogram and repeat scan each trimester is a reasonable approach to evaluate adaptation to pregnancy hemodynamics and to assist in delivery planning. Formation of a multi-disciplinary care delivery plan between the primary OB-GYN, maternal–fetal medicine, cardiology, anesthesia, and nursing is also beneficial in mitigating risk and optimizing outcomes [6, 19].

Although our patient’s echocardiogram findings severely deteriorated from pre-pregnancy values, from the limited history gathered, the patient maintained New York Heart Association Class I or II functional status throughout her antenatal course and had minimal adverse outcomes through delivery and postpartum. A systematic review of various types of inherited cardiomyopathy in pregnancy states that asymptomatic and mildly symptomatic patients have a low risk of adverse events, although long-term studies are noted to be limited [3, 6, 20]. It is possible that since the patient was young, she was better able to compensate against her worsening cardiac function as compared to an older woman in a similar clinical setting. This hypothesis is limited by the patient’s recall bias of her symptomatology and the heterogeneity of clinical manifestations of FDC [21].

This case presented is the first reported case of placement of a WCD in a pediatric patient in the postpartum setting. Due to the elevated risk of ventricular arrhythmia in the postpartum setting, pediatric cardiology recommendations included application of a WCD with re-evaluation postpartum. Duncker et al. demonstrated that adult women with PPCM with severely reduced ejection fraction carry an elevated risk of ventricular tachyarrhythmias that may lead to sudden cardiac death [22]. A retrospective cohort study of WCDs in pediatric patients showed that WCDs were safe and effective in treating these arrhythmias and that compliance was good based on wear time per day [23]. The patient’s mother had previously used a LifeVest before placement of her ICD and was able to assist her daughter in instruction and care, which was greatly beneficial.

Pregnancy in FDC can hold serious morbidity and mortality and ideally requires preconception counseling and close management of prenatal care. Multi-disciplinary management with obstetricians, cardiologists, and anesthesia is crucial in the delivery and postpartum setting to achieve optimal outcomes. Follow up care for patients is crucial to monitor need for ICD placement and conversations with family members on the importance of screening and possible genetic testing.

**Table 1:** Patient’s Echocardiogram Values From Time of Diagnosis to Presentation

Age (years)	LV EF (%)	LV EDD (cm)	Z-score
15 months		3.6 ^a^	
18 months		8.6 ^a^	
2	45	3.5	1.7
2	46	3.6	1.5
3	55	3.7	1.6
4	51	4.1	1.8
5	44	4.1	1.5
6	55	4.5	2.1
7	52	5.0	2.0
7	43	4.4	0.5
8	45	4.8	1.2
8	50	5.3	2.1
9	48	5.3	1.9
10	54	5.6	1.9
11	54	6.4	2.3
13	42	6.3	0.9
14: Three days prior to delivery	“Severely decreased, (EF likely high 20-low 30 s)” ^a^	6.5	1.9
14: 1 month postpartum	Moderately to Severely decreased, < 30% ^b^	6.9	
14: 4 months postpartum	Moderately to Severely decreased ^c^	6.8	

Patient’s echocardiogram values since time of diagnosis

^a^Indicates measurements listed in clinician documentation

^b^Echo report states LVEF is slightly improved when compared with immediate prior study

^c^Unchanged from the study from 1 month postpartum

**Table 2:** Pre-Delivery and Post-Delivery Clinical Data

	Pre-Delivery	On Admission	Postpartum Day 1	Postpartum Day 2	Postpartum Day 3
**Vital Signs**					
Systolic Blood Pressure	147	128	123	101	109
Diastolic Blood Pressure	83	70	81	64	69
Heart Rate	101	90	93	110	106
**Tests**					
EKG	Normal Sinus Rhythm	Normal Sinus Rhythm	No Abnormal Telemetry Events Recorded		
COVID PCR	Negative				
Group B Streptococcus	Positive				
Urine Drug Screen	Negative				
**Lab Values**					
WBC (K/µL)	4.9	5.0	7.5	6.2	8.3
Hemoglobin (g/dL)	10.5	10.4	8.1	8.2	8.8
Hematocrit (%)	33.3	33.0	25.3	26.1	27.9
Platelets (K/µL)	163	178	165	149	162

## Data Availability

The data gathered in this case report were obtained upon review of the patient’s medical record and thus are not publicly available but are available from the corresponding author on reasonable request.

## Abbreviations

CDM: Cardiomyopathy
FDC: Familial dilated cardiomyopathy
LVEF: Left ventricular ejection fraction
ACEI: Angiotensin converting enzyme inhibitors
LVEDD: Left ventricular end diastolic diameter
ICD: Implantable cardioverter defibrillator
MFM: Maternal fetal medicine
WCD: Wearable cardiac defibrillator
PPCM: Peripartum cardiomyopathy
